# Global, regional, and national burden of chlamydial infection: a systematic analysis of incidence, prevalence, deaths, and DALYs with projections to 2046

**DOI:** 10.3389/fpubh.2025.1674277

**Published:** 2025-10-31

**Authors:** Jixu Hu, Yanni Lan, Danyan Zhang, Guipeng Lan, Jiyong Wei

**Affiliations:** ^1^Department of Dermatology and Venereology, The First People’s Hospital of Nanning, The Fifth Affiliated Hospital of Guangxi Medical University, Nanning, China; ^2^Department of Pharmacy, The People’s Hospital of Guangxi Zhuang Autonomous Region and Guangxi Academy of Medical Sciences, Nanning, China; ^3^Department of Bone Surgery, The Eight People’s Hospital of Nanning, Nanning, China; ^4^Department of Orthopedic Surgery, The First People’s Hospital of Nanning, The Fifth Affiliated Hospital of Guangxi Medical University, Nanning, China

**Keywords:** chlamydial infection, global, disease burden, GBD 2021, public health

## Abstract

**Background:**

Chlamydial infection, a major sexually transmitted disease caused by *Chlamydia trachomatis*, imposes a substantial global health burden with uneven distribution. This study aims to quantify its global, regional, and national burden and project trends to 2046.

**Methods:**

Data from the Global Burden of Disease (GBD) 2021 study were analyzed. We estimated 2021 burden metrics, evaluated temporal trends from 1990 to 2021 using estimated annual percentage changes (EAPC), and projected trends for 2022–2046 using an age-period-cohort (APC) model.

**Results:**

In 2021, global incidence was 235.7 million [95% uncertainty intervals (UI): 172.9–334.7 million] with an age-standardized incidence rate (ASIR) of 2902.13/100,000; prevalence was 152.2 million [113.2–213.0 million; age-standardized prevalence rate (ASPR): 1874.56/100,000]. There were 1,033 deaths [683–1,370; age-standardized deaths rate (ASDR): 0.01/100,000] and 163,617 disability-adjusted life-years (DALYs) [116,493–227,160; age-standardized DALYs rate (ASDAR): 2.01/100,000]. Geographic disparities were striking: Southern Sub-Saharan Africa and Central Asia had the highest ASIR, while Western Europe and High-income North America had the lowest. Asia bore the largest absolute burden. Males showed higher incidence rates, while females experienced higher prevalence, deaths, and DALYs. Temporal trends (1990–2021) showed fluctuating case counts and declining age-standardized rates (ASRs), with regional variations. Projections to 2046 indicate divergent sex-specific trends, with rising female ASIR/ASPR but declining absolute cases among males.

**Conclusion:**

Chlamydial infection exhibits marked global disparities, necessitating targeted interventions including region-specific strategies and gender-responsive care to reduce its burden.

## 1 Introduction

Chlamydial infection, caused by *Chlamydia trachomatis*, represents a major global public health challenge and the most commonly reported bacterial sexually transmitted infection worldwide (1). The infection demonstrates a broad clinical spectrum, ranging from asymptomatic cases to severe sequelae including pelvic inflammatory disease, ectopic pregnancy, and infertility in women, and epididymitis and potential fertility implications in men (2). The magnitude of this public health issue is highlighted by WHO estimates indicating approximately 128.5 million new cases of genital chlamydial infections occurred among adults aged 15–49 years globally in 2020 (3). Additionally, chlamydial infection increases susceptibility to HIV acquisition and transmission, further exacerbating its public health impact (4).

Despite its significant health burden, accurate assessment of chlamydial infection’s global impact remains challenging. Substantial underreporting persists, particularly in resource-limited settings with weak surveillance systems (5, 6), and there is a lack of comprehensive, up-to-date, and comparable data across different countries and regions (6). While previous studies and Global Burden of Disease (GBD) reports have provided valuable estimates of chlamydia burden (7), several critical knowledge gaps remain unaddressed.

Compared with previous studies using Global Burden of Disease (GBD) database, our analysis extends beyond descriptive epidemiology in several key ways. First, most existing projections rely on simple extrapolation methods that fail to account for the complex effects of age, period, and birth cohort influences on disease trends. Second, few studies have provided comprehensive, stratified analyses across multiple demographic and geographic dimensions while identifying regions with similar temporal patterns through cluster analysis. Third, there is a lack of long-term projections extending beyond 2030, limiting the ability to inform sustained public health planning. These gaps are particularly concerning given the WHO’s emphasis in its Global Health Sector Strategies on STIs for 2022–2030 on the urgent need for improved surveillance and targeted interventions (8, 9).

To address these limitations, we conducted a comprehensive analysis of the global, regional, and national burden of chlamydial infection using the latest GBD 2021 data. Our study advances the field in several key aspects: First, to our knowledge, this is the first study to apply an age-period-cohort (APC) modeling framework to project chlamydia burden to 2046, providing more demographically robust projections than previous methods. Second, we provide finely stratified analyses by sex, age, Socio-demographic Index (SDI), region, and country, integrating cluster analysis to identify regions with similar temporal trends. Third, our extended projection horizon to 2046 offers policymakers a longer timeframe for intervention planning than previously available.

By addressing these gaps, our study aims to provide comprehensive insights into the past, present, and future burden of chlamydial infection, enabling more effective targeted interventions and resource allocation to reduce the global burden of this infection.

## 2 Methods

### 2.1 Data sources

We sourced data from the GBD 2021 study, which provides comprehensive and comparable estimates of disease burden across different populations (10). This database encompasses data from a wide range of sources, including national surveillance systems, population-based surveys, and hospital records, ensuring a broad coverage for our analysis.

Our analysis included 204 countries and territories that are systematically categorized within the GBD hierarchical geographical framework. These geographical units were organized into 50 GBD regions based on epidemiological similarity and geographical proximity, following the standard GBD regional classification system. Additionally, countries were grouped into 5 SDI quintiles based on their Socio-demographic Index values. The SDI is a composite indicator to quantify the socio-demographic development of geographical regions. The SDI is calculated as the geometric mean of three rescaled components: (1) lag-distributed income per capita, (2) average educational attainment in the population aged 15 years and older, and (3) total fertility rate under age 25. The index ranges from 0 (lowest level of development) to 1 (highest level of development), with regions categorized into five quintiles: low, low-middle, middle, high-middle, and high SDI. This index has been widely used in GBD studies to examine the relationship between socio-economic development and health outcomes. The complete list of countries and their respective GBD regional and SDI classifications is available through the Institute for Health Metrics and Evaluation (IHME) and has been detailed in previous GBD publications.

### 2.2 Ethics statement and data availability

The GBD 2021 study complies with all relevant ethical regulations and received approval from the University of Washington Institutional Review Board. As a secondary analysis of anonymized, aggregated data, this study was exempt from additional ethical approval requirements (10).

All data used in this analysis are publicly available through the Global Health Data Exchange (GHDx) query tool^1^. Specifically, the datasets for chlamydial infection incidence, prevalence, deaths, and disability-adjusted life-years (DALYs) can be accessed using the following parameters: cause = “Chlamydial infection,” measure = “Incidence/Prevalence/Deaths/DALYs,” metric = “Number/Rate,” and year = “1990–2021.” The complete GBD 2021 dataset is also available for download via the Institute for Health Metrics and Evaluation (IHME) website^2^.

### 2.3 Statistical analysis

First, we described global, regional, and national estimates of incidence, prevalence, deaths, and DALYs for 2021, presenting both absolute numbers and age-standardized rates (ASRs per 100,000 population). We stratified the data by sex, age, Socio-demographic Index (SDI) regions, GBD regions, and countries. For the period 1990–2021, we used linear regression to estimate annual percentage changes (EAPC) in ASRs, identifying phases of decreasing/increasing trends. Cluster analysis grouped GBD regions by EAPC values, with results visualized via dendrograms to identify shared trend patterns (11). Finally, we employed an age-period-cohort (APC) model with a maximum likelihood framework to project trends for 2022–2046. This model accounts for the effects of age, time period, and birth cohort on disease burden, enabling more accurate future projections.

We performed analyses in R (version 4.2.3), using dplyr for data manipulation and ggplot2 for visualization.

## 3 Results

### 3.1 The disease burden of chlamydial infection in 2021

In 2021, the global incidence of chlamydial infection was 235,690,238 cases [95% uncertainty intervals (UI): 172,881,033–334,690,522], with an age-standardized incidence rate (ASIR) of 2902.13 (95% UI: 2120.37–4111.26) per 100,000 population. The prevalence reached 152,203,475 (95% UI: 113,239,109–212,994,413) cases, corresponding to an age-standardized prevalence rate (ASPR) of 1,874.56 (95% UI: 1,388.8-2,612.85) per 100,000. A total of 1,033 (95% UI: 683–1,370) deaths were attributed to the disease, with an age-standardized death rate (ASDR) of 0.01 (95% UI: 0.01–0.02) per 100,000. Global DALYs were 163,617 (95% UI: 116,493–227,160), with an age-standardized DALYs rate (ASDAR) of 2.01 (95% UI: 1.43–2.79) per 100,000. For the specific numbers of cases and ASRs of chlamydial infection globally in 1990 and 2021, please refer to Supplementary Tables S1–S4.

Sex-specific analyses revealed distinct disparities: males exhibited higher incidence counts and ASIR, while females bore a greater burden in terms of prevalence, deaths, and DALYs (Supplementary Figure S1; Supplementary Tables S1–S4).

Age-stratified results showed a non-linear trend in disease burden (except for ASDR), characterized by an initial increase with age followed by a decline. In contrast, ASDR demonstrated a consistent upward trend with advancing age (Supplementary Table S2).

At the SDI region level, distinct patterns emerged. Middle SDI regions carried the highest burden for incidence and prevalence, whereas low SDI regions exhibited the highest mortality and DALY rates. High SDI regions consistently showed the lowest burden across all metrics (Supplementary Figure S3; Supplementary Tables S1–S4).

Pronounced geographic disparities were observed at the regional level. Southern Sub-Saharan Africa and Central Asia bore the highest age-standardized rates for incidence and prevalence, while Western Europe and High-income North America had the lowest rates. Asia accounted for the largest absolute number of cases. Detailed regional estimates are provided in Supplementary Figure S4 and Supplementary Tables S1–S4.

**Figure 1:**
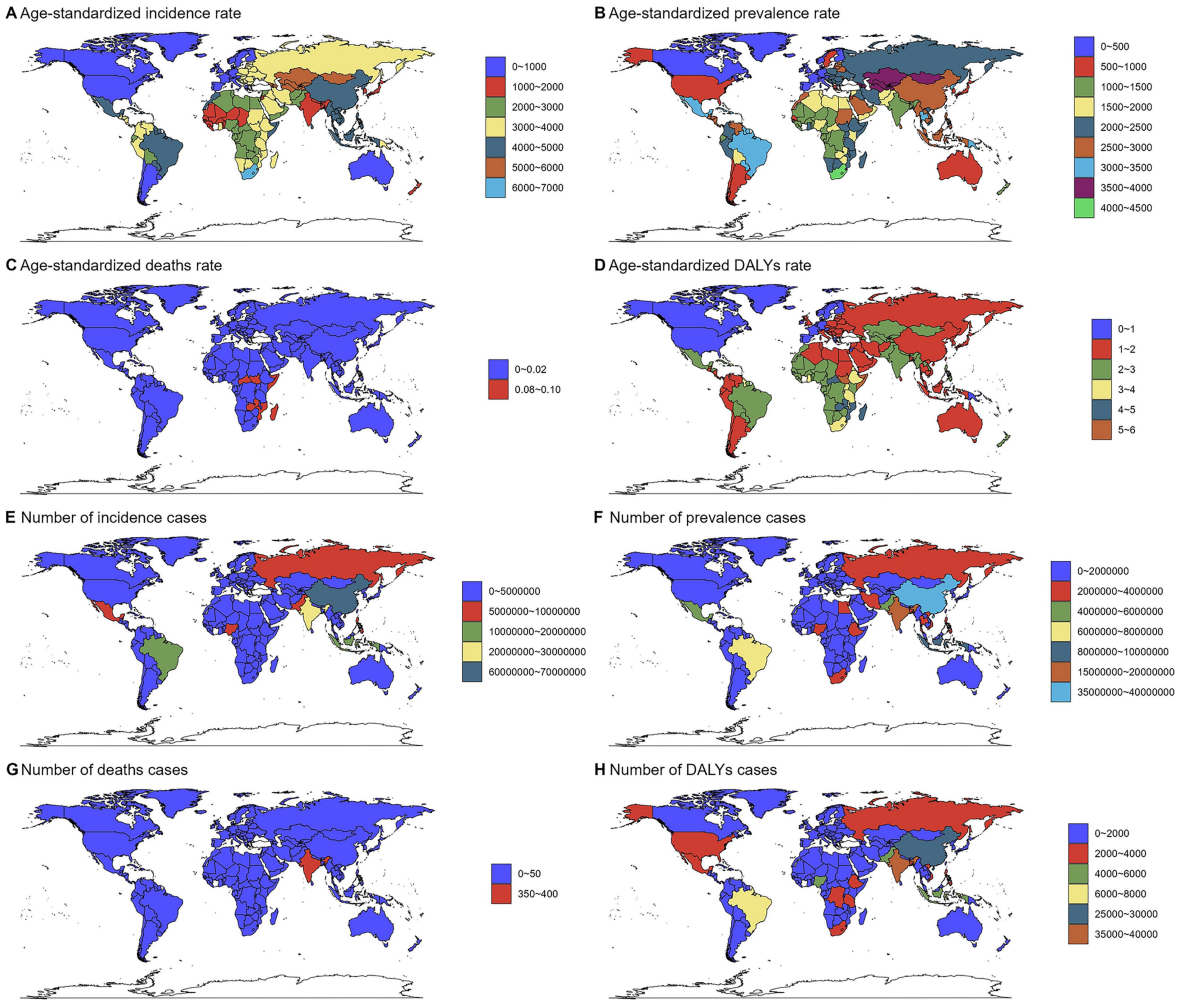
Numbers and age-standardized rates of chlamydial infection-related incidence, prevalence, deaths, and DALYs across countries and territories in 2021. Maps show the geographical distribution of **(A)** age-standardized incidence rate (ASIR, per 100,000 population), **(B)** age-standardized prevalence rate (ASPR, per 100,000), **(C)** age-standardized death rate (ASDR, per 100,000), and **(D)** age-standardized DALYs rate (ASDAR, per 100,000), **(E)** incidence cases, **(F)** prevalence cases, **(G)** deaths cases, and **(H)** DALYs cases. Data were obtained from the Global Burden of Disease Study 2021. The color gradients represent different burden levels. Countries with no data available are shown in gray.

Country-level analysis confirmed significant heterogeneity in disease burden. Selected countries illustrating the range of burden are presented in Figure 1; the complete country-level data are available in Supplementary Tables S1–S4.

### 3.2 Temporal trends in chlamydial infection-related disease burden from 1990 to 2021

**Figure 2:**
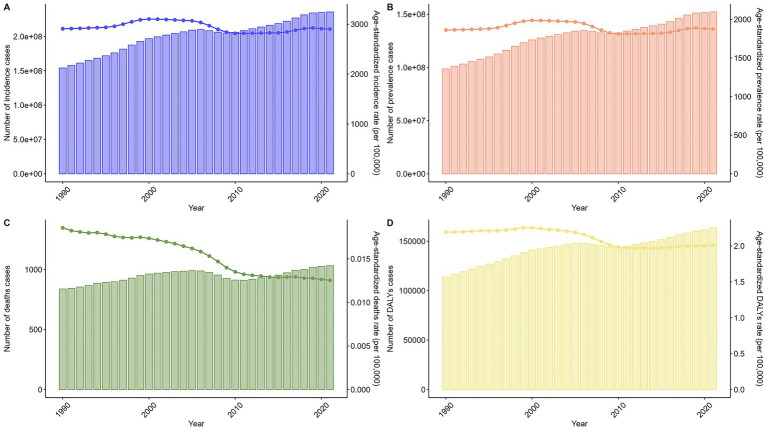
Trends in the numbers and age-standardized rates of chlamydial infection-related incidence, prevalence, deaths, and DALYs globally from 1990 to 2021. Line graphs show temporal patterns in **(A)** age-standardized incidence rate (ASIR, per 100,000 population), **(B)** age-standardized prevalence rate (ASPR, per 100,000), **(C)** age-standardized death rate (ASDR, per 100,000), and **(D)** age-standardized DALYs rate (ASDAR, per 100,000). Bar charts show temporal patterns in **(A)** incidence cases, **(B)** prevalence cases, **(C)** deaths cases, and **(D)** DALYs cases.

Globally, case counts for incidence, prevalence, deaths, and DALYs showed a trend of initial increase, subsequent decrease, and final increase. Age-standardized rates exhibited fluctuating downward trends (Figure 2). For the trends for specific numbers of cases and ASRs of chlamydial infection globally from 1990 to 2021, please refer to Supplementary Tables S1–S4.

Sex-specific and age-specific trends largely mirrored the overall global pattern (Supplementary Figures S5, S6). Trends across SDI regions were generally consistent with the total population, except for high SDI regions which showed distinct patterns for ASIR and ASPR (Supplementary Figure S7).

**Figure 3:**
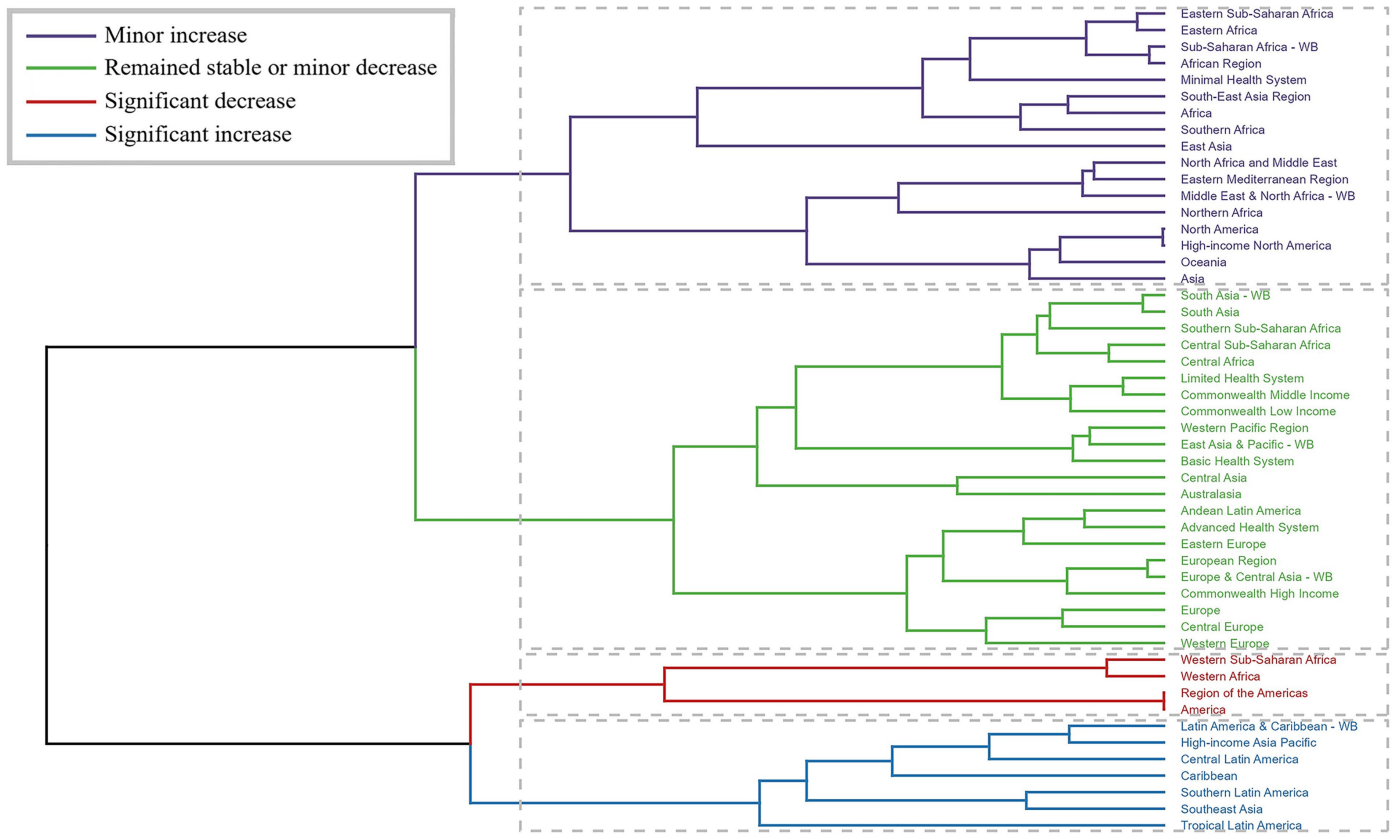
Results of cluster analysis based on the EAPC values of the chlamydial infection-related age-standardized rates for incidence, prevalence, deaths, and DALYs from 1990 to 2021. Dendrogram illustrates the clustering of GBD regions based on similarity in estimated annual percentage change (EAPC) patterns. Colors indicate different trend categories: blue for significant increase, red for significant decrease, yellow for minor increase, and green for remained stable or minor decrease. The clustering was performed using Ward’s method with Euclidean distance, revealing regions with similar temporal evolution patterns.

Cluster analysis of GBD regions based on EAPC values revealed heterogeneous temporal patterns (Figure 3). Significant increases in rates were observed in several regions including Latin America & Caribbean and High-income Asia Pacific, while decreases were noted in Western Sub-Saharan Africa and Western Africa.

**Figure 4:**
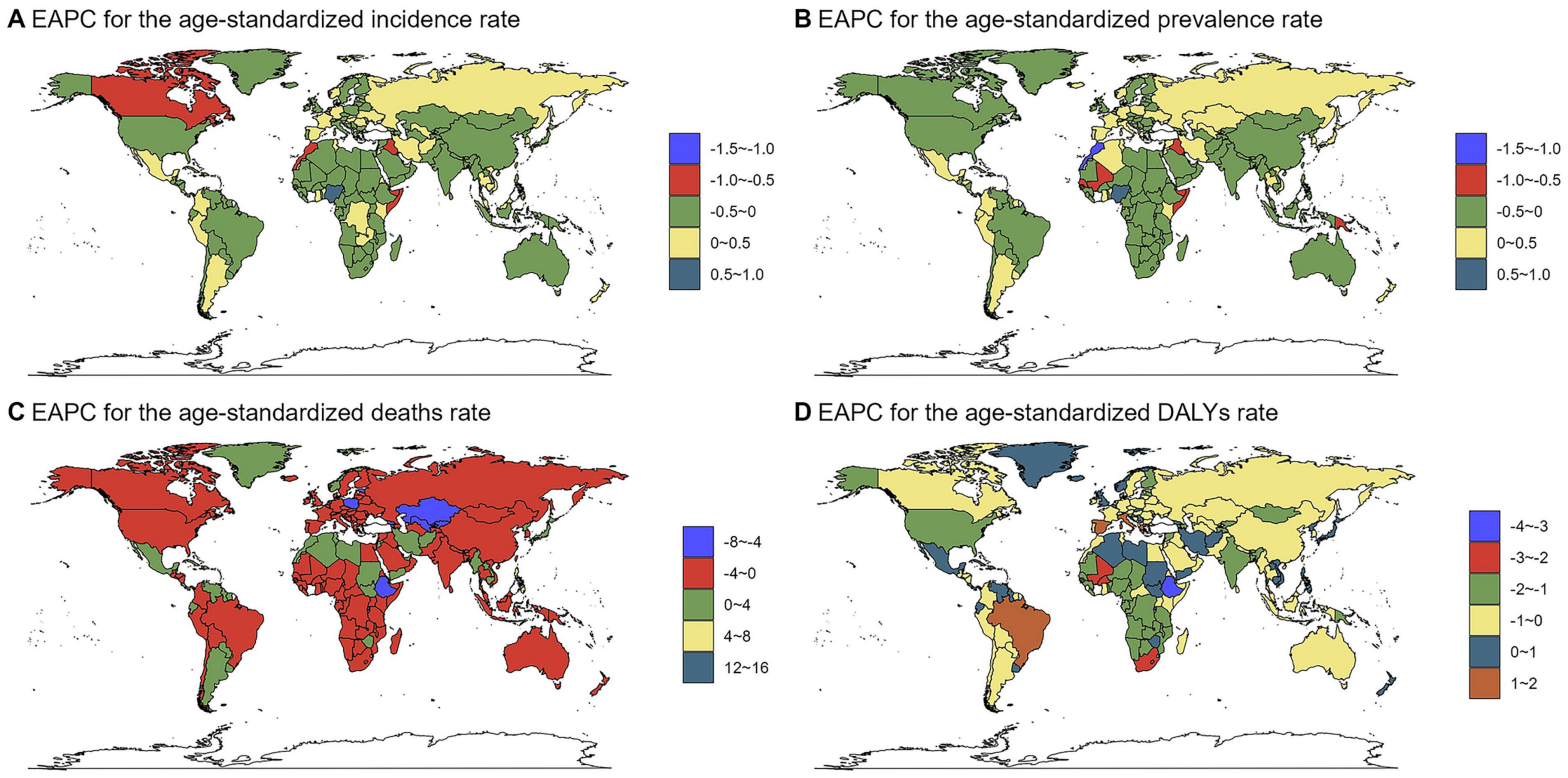
The EAPC of chlamydial infection-related ASRs from 1990 to 2021. Maps display the geographical distribution of estimated annual percentage change (EAPC) for **(A)** age-standardized incidence rate (ASIR, per 100,000 population), **(B)** age-standardized prevalence rate (ASPR, per 100,000), **(C)** age-standardized death rate (ASDR, per 100,000), and **(D)** age-standardized DALYs rate (ASDAR, per 100,000). The color scale represents different ranges of EAPC values.

Country-level trends varied markedly. Nigeria showed the most substantial increases in ASIR and ASPR, whereas the Marshall Islands experienced the sharpest declines. The most pronounced changes in mortality (ASDR) and DALY rates (ASDAR) were observed in the Northern Mariana Islands (decrease) and Mauritius (increase), and Italy (increase) and Ethiopia (decrease), respectively. The complete country-level trend analysis is provided in Supplementary Tables S1–S4 and visualized in Figure 4.

### 3.3 The predicted results from 2022 to 2046

Projections indicate varied trends in incidence, prevalence, deaths, and DALYs for both sexes. For females, the number of incidence cases is expected to change from 221,915,443 (2022) to 123,932,521 (2046). Prevalence cases are projected to shift from 195,063,930 in 2022 to 108,612,259 in 2046. Deaths are anticipated to change from 2,033 in 2022 to 949 in 2046, and DALYs cases from 205,587 in 2022 to 112,554 in 2046. For ASRs (per 100,000 population): ASIR is projected to increase from 769,3.81 in 2022 to 853,0.26 in 2046; ASPR will rise from 675,3.10 to 746,7.60; ASDR will decline from 0.051 to 0.047; and ASDAR will increase from 6.93 to 7.45.

**Figure 5:**
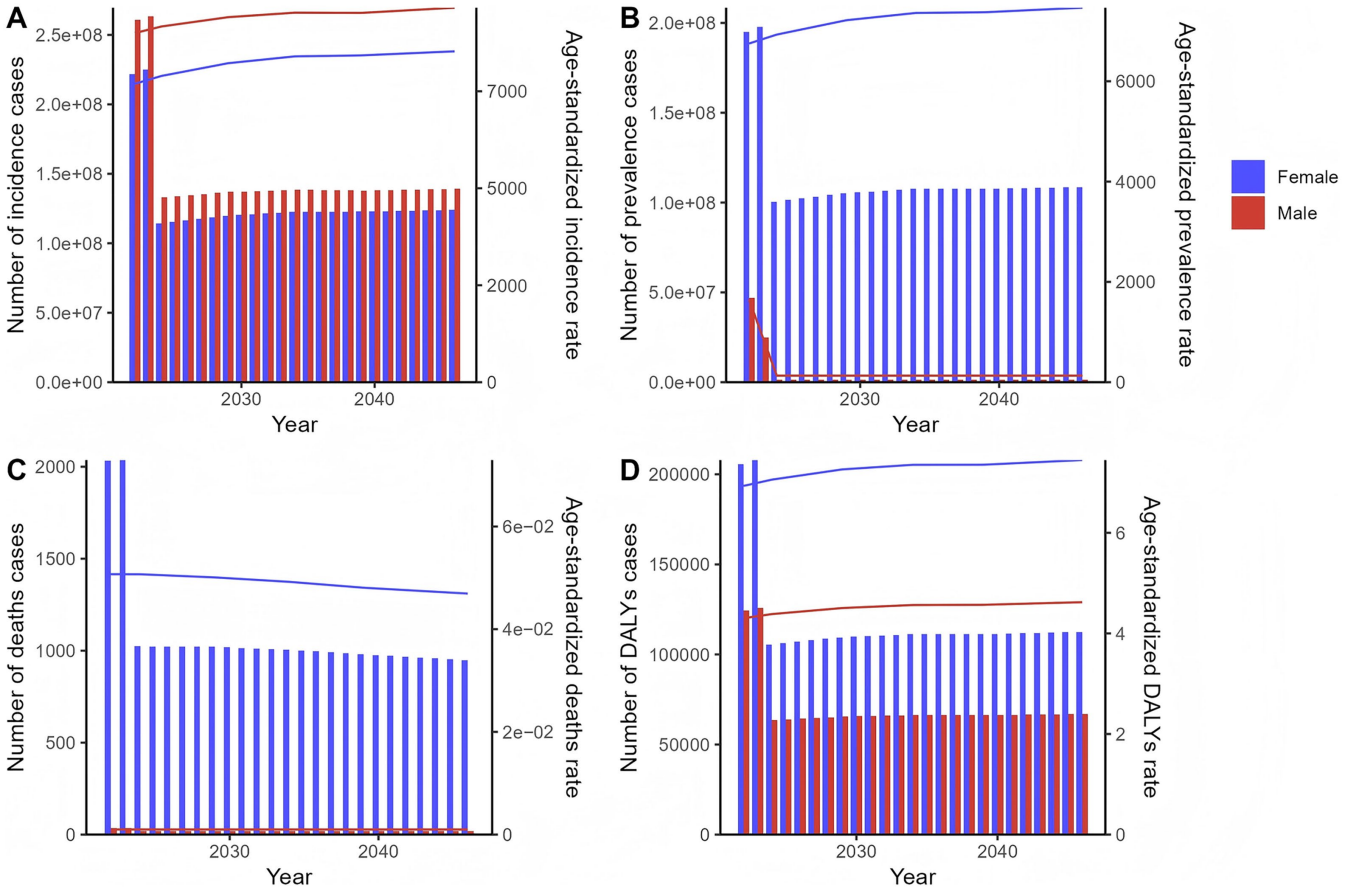
The predicted results in the chlamydial infection-related numbers and age-standardized rates of incidence, prevalence, deaths, and DALYs by sex globally from 2022 to 2046 of the age-period-cohort model. Line graphs show projected trends for males (blue) and females (red) for **(A)** age-standardized incidence rate (ASIR, per 100,000 population), **(B)** age-standardized prevalence rate (ASPR, per 100,000), **(C)** age-standardized death rate (ASDR, per 100,000), and **(D)** age-standardized DALYs rate (ASDAR, per 100,000). Bar charts show temporal patterns in **(A)** incidence cases, **(B)** prevalence cases, **(C)** deaths cases, and **(D)** DALYs cases. The projections were generated using an age-period-cohort model based on historical data from 1990 to 2021.

For males, the number of incidence cases is expected to decrease from 260,731,487 (2022) to 139,177,812 (2046). Prevalence cases are projected to decline from 47,026,616 in 2022 to 1,406,644 in 2046. Deaths are anticipated to drop from 35 in 2022 to 18 in 2046, and DALYs cases from 124,384 in 2022 to 66,897 in 2046. For ASRs (per 100,000 population): ASIR will show a complex trend, fluctuating around a high level, from 9,002.02 in 2022 to 9,655.93 in 2046; ASPR will decrease from 1,649.28 to 130.49; ASDR will remain constant at 0.001 throughout the period; and ASDAR will increase from 4.30 to 4.62 (Figure 5; Supplementary Table S5).

## 4 Discussion

This study provides a comprehensive analysis of the global, regional, and national burden of chlamydial infection, including incidence, prevalence, deaths, and DALYs, with projections through 2046. The findings reveal striking disparities in disease burden across geographic regions, socioeconomic strata, and demographic groups, alongside dynamic temporal trends and divergent future projections. These results underscore the complex interplay of biological, behavioral, and structural factors shaping chlamydial infection epidemiology and highlight the need for targeted public health interventions to address inequities.

The observed global burden of chlamydial infection reflects longstanding patterns of health disparities documented in previous research. Geographic variations, with high burdens in Southern Sub-Saharan Africa, Central Asia, and parts of Southeast Asia, and lower burdens in Western Europe and High-income North America, align with broader global trends in sexually transmitted infections (STIs) (12, 13). These disparities stem from multiple factors, including limited healthcare access, inadequate STI surveillance, and socioeconomic inequalities. For instance, Sub-Saharan Africa’s high age-standardized incidence and prevalence rates reflect challenges such as underfunded public health systems, low rates of routine screening, and cultural stigmatization of STIs, which hinder timely diagnosis and treatment (14, 15). A recent WHO report highlights that despite global targets for STI reduction, progress remains uneven, and many high-burden countries continue to face significant barriers to achieving these goals due to funding shortfalls and system-level challenges (16). Conversely, the lower burden in high-income regions likely reflects investments in sexual health education, widespread screening programs (annual chlamydia testing for sexually active youth), and easy access to antibiotics, which reduce transmission and sequelae (17, 18).

Sex-specific differences in disease burden, with males exhibiting higher incidence but females bearing greater prevalence, deaths, and DALYs, are consistent with biological and behavioral realities of chlamydial infection. Females are anatomically more susceptible to asymptomatic infection and ascending genital tract involvement, which increases the risk of long-term sequelae such as pelvic inflammatory disease (PID) and infertility (19, 20). This susceptibility, compounded by lower rates of routine screening in some settings, may explain their higher prevalence and DALYs (21). Males, while more likely to report symptomatic urethritis, often seek treatment earlier, reducing their risk of chronic complications (22). These findings reinforce the need for gender-responsive interventions, such as targeted screening campaigns for females and expanded access to care for males in underserved areas.

Age-stratified trends, characterized by a non-linear pattern in disease burden (peaking in sexually active age groups) and rising mortality with advanced age, align with the epidemiology of chlamydial infection. Young adults (15–24 years) face elevated risk due to higher rates of unprotected sex, multiple partners, and limited engagement with healthcare systems (23, 24). The upward trend in age-standardized death rates among older adults may reflect delayed diagnosis of complications (ectopic pregnancy, disseminated infection) or comorbidities such as HIV, which exacerbate outcomes (25, 26). These patterns emphasize the importance of age-tailored prevention strategies, including school-based sexual health education and geriatric sexual health screenings.

Socioeconomic gradients in disease burden, as captured by SDI strata, further highlight the role of structural determinants in shaping chlamydial infection dynamics. Middle SDI regions exhibit high incidence and prevalence, potentially due to a “transition effect”: increasing sexual risk behaviors (delayed marriage, higher partner counts) alongside incomplete expansion of healthcare infrastructure to address emerging needs (27, 28). Low SDI regions, meanwhile, bear the highest mortality and DALYs, reflecting limited access to antibiotics and inadequate management of sequelae (29). High SDI regions’ favorable outcomes underscore the impact of robust healthcare systems, including universal screening and affordable treatment (30). These findings support the broader literature linking STI burden to socioeconomic development and reinforce the need for investments in health systems strengthening in low- and middle-income settings.

Temporal trends from 1990 to 2021, with global case counts fluctuating and age-standardized rates declining overall, reflect the combined effects of intervention efforts and changing population dynamics. Declines in age-standardized rates may partly stem from expanded screening and treatment programs, such as those implemented in Europe and North America since the early 2000s (31, 32). However, regional heterogeneities, including increases in Latin America and decreases in Western Sub-Saharan Africa, highlight the variable success of these interventions. For example, gains in Western Sub-Saharan Africa may be attributed to targeted HIV/STI integration programs, which leverage existing HIV infrastructure to address chlamydia (33), while increases in Latin America could reflect improved surveillance capturing previously underreported cases (34).

Country-specific trends, such as rising incidence in Nigeria and declining rates in the Marshall Islands, underscore the influence of local policies and population characteristics. Nigeria’s increasing burden may relate to rapid urbanization, which often correlates with higher sexual risk behaviors, and gaps in youth-focused sexual health programs (35). In contrast, the Marshall Islands’ decline could reflect successful community-based interventions, such as school-based education and outreach to at-risk groups (36). These variations emphasize the need for context-specific strategies rather than one-size-fits-all approaches.

Projections through 2046, with divergent trends by sex and region, highlight the potential impact of future public health investments. For females, rising age-standardized incidence and prevalence rates may signal unmet needs in sexual health care, particularly in regions where gender disparities in healthcare access persist (37). For males, declining absolute cases could reflect sustained efforts to engage men in screening and treatment, though high age-standardized rates in some regions warrant continued attention (38). These projections align with models predicting that without accelerated intervention, STI burdens will remain elevated in low-resource settings, while high-income regions may see modest declines (39).

Notably, the divergent trends between sexes, increasing female age-standardized incidence and prevalence rates (ASIR/ASPR) versus declining male case counts, may be attributed to a combination of biological susceptibility, healthcare-seeking behavior, and diagnostic practices. Biologically, women are more susceptible to persistent or recurrent chlamydial infections due to anatomical factors (cervical ectopy) and a higher likelihood of asymptomatic presentation, which can lead to untreated infections and subsequent complications such as PID and infertility (19, 20). This may contribute to sustained high prevalence and incidence rates in women. In contrast, men are more likely to exhibit symptomatic infections (urethritis), prompting earlier healthcare-seeking and treatment, thereby reducing transmission and overall case counts over time (22). Furthermore, disparities in healthcare access and diagnostic practices may exacerbate these trends. In many regions, women face structural barriers to sexual health services, including stigma, cost, and limited availability of screening programs (37). Even when services are available, cultural factors may discourage women from seeking timely care. Conversely, symptomatic men may be more likely to engage with healthcare systems, especially in settings where STI clinics are accessible. Additionally, global health programs have historically focused on maternal and reproductive health, potentially leading to better detection and reporting of female cases, though not necessarily better outcomes. These factors collectively contribute to the observed divergence in sex-specific trends. Finally, the rising female ASIR/ASPR in some regions may also reflect improvements in diagnostic sensitivity and public health screening efforts targeting women, such as prenatal or family planning clinics (18, 21). As screening expands, previously undetected cases are identified, artificially elevating incidence rates in the short term. In the long term, however, effective screening and treatment should lead to reduced transmission and lower rates. The persistence of high rates in women underscores the need for enhanced secondary prevention and partner management strategies.

This study’s strengths include its use of the Global Burden of Disease (GBD) dataset, which provides standardized, comparable estimates across 204 countries and territories (10). The application of the age-period-cohort (APC) model for projections enhances the robustness of future trends by accounting for demographic shifts and temporal effects (40). However, limitations must be acknowledged. First, underreporting remains a challenge, particularly in regions with weak surveillance systems, potentially underestimating true burden (41). Second, the APC model’s assumptions, including stable trends in risk factors, may not account for unforeseen events (pandemics, policy changes) that could alter projections (42). Finally, the analysis focuses on chlamydia alone, without exploring its interaction with other STIs (gonorrhea, HIV), which can amplify transmission and severity (43).

This study advances the existing literature on the global burden of chlamydial infection in several important respects. While earlier studies have provided valuable snapshots of chlamydia epidemiology (12, 13, 39), our use of the APC model for long-term projections is a significant methodological innovation. The APC framework allows us to disentangle the effects of aging, temporal trends, and birth cohort influences, thereby producing more reliable and interpretable projections than those derived from simple time-series extrapolations. For example, our cohort-based insights can help identify generations that may be at higher risk due to historical changes in sexual behavior or public health policies. Furthermore, our integration of cluster analysis with EAPC estimation enables the identification of groups of countries and regions with similar evolutionary trends, which can inform targeted regional strategies. Finally, our detailed stratification and extended projection period provide a more comprehensive basis for understanding demographic and geographic disparities and for planning long-term public health responses. Together, these elements enhance the practical utility of our findings for global health planning and resource allocation.

These findings have critical implications for public health policy. First, efforts to reduce disparities must prioritize high-burden regions, such as Southern Sub-Saharan Africa and Central Asia, through investments in healthcare infrastructure, including point-of-care testing and affordable antibiotics (44). Second, gender-responsive strategies are needed: for females, expanding access to gynecological care and addressing barriers to screening (stigma, cost); for males, increasing awareness of asymptomatic infection and encouraging partner notification (45). Third, integrating chlamydia prevention with existing HIV and reproductive health programs could improve efficiency, as demonstrated by successful models in Sub-Saharan Africa (46). The WHO’s recently updated guidelines on STI management reinforce the importance of such integrated, people-centered approaches to service delivery (47). Fourth, long-term investments in vaccine development, though challenging given chlamydia’s antigenic diversity, could complement existing strategies; recent advances in antigen discovery and platform technology offer renewed hope for future vaccines (48). Finally, strengthening surveillance systems, particularly in low- and middle-income countries, is essential to better track trends and evaluate intervention impact, a point strongly emphasized in the 2022–2030 global strategies (49).

## 5 Conclusion

In conclusion, chlamydial infection remains a significant global public health challenge, with disparities shaped by geography, socioeconomic status, and gender. While progress has been made in some regions, sustained, context-specific efforts are needed to reduce burden and achieve equitable outcomes. This study’s findings provide a roadmap for targeting interventions, emphasizing the urgency of addressing structural inequities and investing in sexual health for all.

## Data Availability

The original contributions presented in the study are included in the article/Supplementary material, further inquiries can be directed to the corresponding authors.

## Footnotes

^1^ http://ghdx.healthdata.org/gbd-results-tool

^2^ http://ghdx.healthdata.org/gbd-2021

## Supplementary material

SUPPLEMENTARY FIGURE S1 Numbers and age-standardized rates of chlamydial infection related incidence, prevalence, deaths, and DALYs for both sex in 2021. Line graphs compare the burden between males (blue) and females (red) for **(A)** age-standardized incidence rate (ASIR, per 100,000 population), age-standardized prevalence rate (ASPR, per 100,000), age-standardized death rate (ASDR, per 100,000), and age-standardized DALYs rate (ASDAR, per 100,000). Bar charts compare the burden between males (blue) and females (red) for **(B)** incidence cases, prevalence cases, deaths cases, and DALYs cases.

SUPPLEMENTARY FIGURE S2 Numbers and age-standardized rates of chlamydial infection related incidence, prevalence, deaths, and DALYs for different age groups in 2021. Line graphs display age-specific patterns for **(A)** age-standardized incidence rate (ASIR, per 100,000 population), age-standardized prevalence rate (ASPR, per 100,000), age-standardized death rate (ASDR, per 100,000), and age-standardized DALYs rate (ASDAR, per 100,000). Bar charts display age-specific patterns for **(B)** incidence cases, prevalence cases, deaths cases, and DALYs cases.

SUPPLEMENTARY FIGURE S3 Numbers and age-standardized rates of chlamydial infection related incidence, prevalence, deaths, and DALYs for different SDI region in 2021. Line graphs compare burden across SDI regions for **(A)** age-standardized incidence rate (ASIR, per 100,000 population), age-standardized prevalence rate (ASPR, per 100,000), age-standardized death rate (ASDR, per 100,000), and age-standardized DALYs rate (ASDAR, per 100,000). Bar charts compare burden across SDI regions for **(B)** incidence cases, prevalence cases, deaths cases, and DALYs cases.

SUPPLEMENTARY FIGURE S4 Numbers and age-standardized rates of chlamydial infection related incidence, prevalence, deaths, and DALYs for different GBD region in 2021. Line graphs compare burden across GBD regions for **(A)** age-standardized incidence rate (ASIR, per 100,000 population), age-standardized prevalence rate (ASPR, per 100,000), age-standardized death rate (ASDR, per 100,000), and age-standardized DALYs rate (ASDAR, per 100,000). Bar charts compare burden across GBD regions for **(B)** incidence cases, prevalence cases, deaths cases, and DALYs cases.

SUPPLEMENTARY FIGURE S5 Trends in the numbers and age-standardized rates of chlamydial infection-related incidence, prevalence, deaths, and DALYs globally by sex from 1990 to 2021. Trends in the numbers and age-standardized rates of chlamydial infection-related incidence, prevalence, deaths, and DALYs globally by age groups from 1990 to 2021. Line graphs show temporal patterns for different sexes for **(A)** age-standardized incidence rate (ASIR, per 100,000 population), age-standardized prevalence rate (ASPR, per 100,000), age-standardized death rate (ASDR, per 100,000), and age-standardized DALYs rate (ASDAR, per 100,000), **(B)** incidence cases, prevalence cases, deaths cases, and DALYs cases.

SUPPLEMENTARY FIGURE S6 Trends in the numbers and age-standardized rates of chlamydial infection-related incidence, prevalence, deaths, and DALYs globally by age groups from 1990 to 2021. Line graphs show temporal patterns for different age groups for **(A)** age-standardized incidence rate (ASIR, per 100,000 population), age-standardized prevalence rate (ASPR, per 100,000), age-standardized death rate (ASDR, per 100,000), and age-standardized DALYs rate (ASDAR, per 100,000), **(B)** incidence cases, prevalence cases, deaths cases, and DALYs cases.

SUPPLEMENTARY FIGURE S7 Trends in the numbers and age-standardized rates of chlamydial infection-related incidence, prevalence, deaths, and DALYs globally by SDI region from 1990 to 2021. Trends in the numbers and age-standardized rates of chlamydial infection-related incidence, prevalence, deaths, and DALYs globally by age groups from 1990 to 2021. Line graphs show temporal patterns for different SDI regions for **(A)** age-standardized incidence rate (ASIR, per 100,000 population), age-standardized prevalence rate (ASPR, per 100,000), age-standardized death rate (ASDR, per 100,000), and age-standardized DALYs rate (ASDAR, per 100,000), **(B)** incidence cases, prevalence cases, deaths cases, and DALYs cases.
